# Understanding the Perceived Threat of the Risk of Graft Rejections: A Middle-Range Theory

**DOI:** 10.1177/2333393614563829

**Published:** 2015-01-21

**Authors:** Anna Forsberg, Annette Lennerling, Isabell Fridh, Veronika Karlsson, Madeleine Nilsson

**Affiliations:** 1 Lund University, Lund, Sweden; 2 Skåne University Hospital, Lund, Sweden; 3 Sahlgrenska University Hospital, Gothenburg, Sweden; 4 University of Gothenburg, Göteborg, Sweden; 5 University of Borås, Borås, Sweden; 6 University of Skövde, Skövde, Sweden

**Keywords:** nursing, transplantation, graft rejection, middle-range theory, organ transplant recipients, perceived threat

## Abstract

From a clinical viewpoint, graft rejection is one of the greatest threats faced by an organ transplant recipient (OTR). We propose a middle-range theory (MRT) of Perceived Threat of the Risk of Graft Rejection (PTRGR) as a contribution to the practice of transplant nursing. It could also apply to the detection of risky protective behavior, that is, isolation, avoidance, or non-adherence. The proposed MRT covers the following concepts and the relationship between them: transplant care needs, threat reducing interventions, intervening variables, level of PTRGR, protective strategies, and evidence-based practice. Parts of this theory have been empirically tested and support the suggested relationship between some of the concepts. Further tests are needed to strengthen the theoretical links. The conceptual framework might serve as a guide for transplant nurses in their efforts to promote post-transplant health and reduce threat-induced emotions.

## Introduction

Solid organ transplantation is an established and successful treatment for critically ill patients. For many patients with severe organ failure, it is the only option for survival. After transplantation, the treatment consists of medications intended to suppress the immune system of organ transplant recipients (OTRs) to prevent graft rejection. According to clinical practice, the transplanted patient is required to submit to an extensive follow-up program for the purpose of early identification of graft rejection, infection, or other complications, especially during the first post-operative year. The patient is also expected to develop an adequate self-care capacity regarding medication, observing signs of graft rejection as well as dealing with new demands and changes in daily life.

A common intervention in different transplant settings worldwide is to educate the patients in “patient awareness,” before as well as after discharge from the transplant unit. The aim of this is to provide OTRs with tools and strategies on how to avoid exposure to risks that could lead to infections or graft rejection. The key message in most awareness training is that OTRs should live as normally as possible, in spite of having received a transplant. The life of the OTR is thus transformed from one of suffering from a life-threatening illness to something more healthy when compared with the situation before the transplantation, but with the underlying necessity of undergoing lifelong immunosuppressive treatment and follow-up because of the still chronic condition. When OTRs are asked about what they fear most, the most common response is graft rejection. The Perceived Threat of the Risk of Graft Rejection (PTRGR) is prominent in the lives of OTRs (Nilsson, Persson, & Forsberg, 2008). The threat of graft rejection is not just a potential threat but a threat with a rather high risk of occurring. If it occurs, it might irreversibly harm the transplanted organ. The OTRs are instructed on how to recognize signs and symptoms of graft rejection before being discharged from the transplant unit.

Within the field of transplant nursing, there is a distinct lack of specific transplant-related nursing theories that identify and express the key ideas about the essence of transplant nursing practice. The already existing nursing theories such as Neuman’s system theory on stressors (Neuman & Fawcett, 2011) or the theory of transition developed by Meleis (2007) might be useful in some cases. However, they are too general in their content. The specific issue of organ transplantation and graft rejection demands more context-specific theories to guide evidence-based nursing practice. Transplant-related nursing interventions must be directed to reduce the OTRs’ fear of graft rejection, and yet maintain the respect for the risk and the necessity of adherence to the immunosuppressive medication regimen. There are three requirements for the transplant professionals: to be proactive, reactive, and preventive. The phenomenon is in itself complicated, and the need for a theory that explains and deals with this clinically important issue in transplant nursing therefore becomes essential.

### Rationale

The rationale behind our efforts to develop this middle-range theory (MRT) is based on vast clinical experience that clearly shows that the threat of the risk for graft rejection is prominent in the lives of OTRs. Together with infection, graft rejection is viewed as the most common threat for these patients. Previous studies (Forsberg, Möller, & Bäckman, 2000; Nilsson, 2010; Nilsson, Forsberg, Bäckman, Lennerling, & Persson, 2011; Nilsson, Forsberg, Lennerling, & Persson, 2013; Nilsson et al., 2008) indicate that the threat of the risk for graft rejection might have a negative impact on the patients’ everyday life. In spite of this, research into patients’ perceptions of their experience of graft rejection or the threat of it has been a neglected field. Although there are great advances in exploring the physiological mechanisms behind graft rejection and the biomedical treatment of the immunological processes causing graft rejection (Ekberg et al., 2009, Frei et al., 2010), key ideas on the essence of nursing practice in relation to graft rejection need to be identified and expressed. The OTRs’ perceptions and experiences, as well as consequences, such as Health-Related Quality of Life (HRQoL) in relation to the threat of the risk for graft rejection, have been poorly understood. For example, how do OTRs in various ages perceive the threat of the risk for graft rejection? No validated domain-specific instrument was ever used to measure this perceived threat among OTRs receiving various types of solid organs, before our group actually developed one (Nilsson et al., 2011). The different characteristics of the threat of the risk for graft rejection were rarely described and the absence of systematic and structured measurements hampered the possibility to make any comparisons between groups of OTRs to evaluate the effects of various interventions.

The rationale behind this MRT is to support nursing care for OTRs experiencing the threat of the risk for graft rejection and who also show negative psychological reactions. Altogether, this knowledge is important for the health care professionals to improve existing strategies of relieving the condition. There is also a need to develop methods that support those patients where the HRQoL as well as the pattern of daily life is affected. The concept of threat is critical to nurses who are faced with the challenge of caring for those experiencing threat-induced emotions. Regardless of whether the threat is perceived or real, as in the case of a biopsy-proven graft rejection, this threat induces various negative emotional responses and has deep psychophysiological and social consequences (Nilsson, 2010; Nilsson et al., 2011; Nilsson et al., 2013; Nilsson et al., 2008). It is therefore important that all health care professionals cooperate in the field of organ transplantation to acknowledge the patients’ reasoning and be able to provide proper individualized intervention.

The foremost rational for this MRT is to create a firm base of knowledge from the OTRs’ perspective that will be useful in both the care and training of patients that suffer from the PTGR[Perceived Threat of the Risk of Graft Rejection]. It will also serve as a basis for future longitudinal and interventional studies. To summarize, the aim of this article is to propose a MRT dealing with the PTRGR among solid OTRs.

### Previous Research

For many years, our research has focused on the specific event of graft rejection within the context of solid organ transplantation. The results of these studies (Forsberg et al., 2000; Nilsson, 2010; Nilsson et al., 2011; Nilsson et al., 2013; Nilsson et al., 2008) have encouraged us to develop a theory that assists organ transplant nurses understand practice in a more complete and insightful way. Before we began to explore extensively the PTRGR, few nursing-related studies had been performed among OTRs, which meant that the perceptions underlying this threat were poorly understood. In previous studies, OTRs were asked to say what they most feared or most stressed them. The most common response was graft rejection (De Vito Dabbs et al., 2004; Gubby, 1998; Kong & Molassiotis, 1999; Luk, 2004). In the study by Fallon, Gould, and Wainwright (1997), the aim was to identify specific stress factors among kidney transplanted recipients at different time intervals after transplantation (6 months, 1–5 years, and more than 5 years). Regardless of time intervals, the most common factor was the possible risk of rejection. It was shown that fear of rejection decreased with time following transplantation but remained the main stress factor. Kong and Molassiotis (1999) found similar results.

Also in qualitative studies, the risk of rejection has been found to be the most stressful event among OTRs. Luk (2004) collected data via interviews. Kidney transplant recipients with an average time of 5.5 years since transplantation were asked what they found most difficult. Most of them talked about the risk of rejection. In a study by De Vito Dabbs et al. (2004) where lung transplant recipients were interviewed, it was found that after transplantation, patients were striving to live normally. Striving to live normally was the core process involving symptom experience and interpretation associated with rejection. The development of rejection marked the beginning of the vulnerability stage. When rejection occurred, recipients expressed surprise and disappointment. Forsberg et al. (2000) showed that liver transplant recipients at follow-up 1 year after transplantation also experienced the threat of graft rejection as alternating from being something of no specific significance to fear of death. These feelings involved being constantly aware of their bodies, being continually in fear, experiencing an invisible threat, and of being failed or simply “let down” by their bodies.

Gubby (1998) involved 30 liver transplant recipients and concluded that the threat of graft rejection was the single most significant stress factor. Early studies indicate that the threat of the risk for graft rejection has a negative impact on the patients’ everyday life (House, Dubovsky, & Penn 1983; Surman, 1989; Surman, Dienstag, Cosimi, Chauncey, & Russels, 1987). Despite the limited research on OTRs’ perceptions of experiences of graft rejection or the threat, we think it is relevant to summarize the present scientific knowledge and to consider its relevance to transplant nursing.

## Method

The elements and strategies for theory building proposed by Walker and Avant (2011) were chosen relying on the definition of a theory “as an internally consistent group of related statements that present a systematic view of a phenomenon that is useful for description, explanation, prediction and prescription or control” (p. 61). Sets of definitions were developed that are specific to concepts in the theory. During theory development, we moved freely among three approaches to theory building:

- *Derivation*, where we transposed and redefined the concept threat from a concept analysis performed by Ritchie (2004) and the concept harm from a description by Carpenter (2005).
- *Synthesis*, where we constructed new statements and a premature theory based on our literature review and the findings of observations made in empirical research (Forsberg et al., 2000; Nilsson 2010; Nilsson et al., 2011; Nilsson et al., 2013; Nilsson et al., 2008). We used synthesis both to name clusters in one of our factor analyses and to name the categories in a phenomenographic qualitative data analysis.
- *Analysis*, where we examined the relationship of some parts of the theory and tried to improve accuracy and relevance of the knowledge gained.

Although we moved back and forth between the three approaches, we will present the results from each step separately to present a clearer picture of each one.

Different sources of data were used. First we explored existing literature, and whether or not it was research based or linked to any common conceptual or theoretical framework. No such framework was identified although the common theme was that the threat of risk of graft rejection was perceived as something negative and highly stressful, as reported in the “Introduction” section. No state-of-the-art articles, relevant to nursing, were available regarding graft rejection. We acknowledged the data to which we had direct access that was obtained from several of our empirical studies, and finally we joined our personal judgments on how to best approach the theory building process. We weighed the current knowledge attained through empirical studies and literature reviews, and have now tried to synthesize it by means of an MRT applicable to the field of transplant nursing.

## Research Ethics

The MRT is based on the studies approved by the central ethical review board (Dnr 569-07).

## Results

### Concept and Statement Derivation

The definitions given below are based on a subjective perspective of the OTR where the individual’s experience is the most important. The purpose of this MRT of PTRGR is to assist transplant nursing in (a) caring for OTRs suffering from threat-induced emotions because of the constant risk of graft rejection that negatively affect and limit their everyday life and HRQoL and (b) detecting risky, protective behavior adopted by the OTR to prevent graft rejection or manage the sense of fear, that is, isolation, avoidance, or non-adherence.

The concept PTRGR was derived primarily from the three references below. According to O’Byrne (2008), risk might be defined as being exposed to the likelihood of a negative event and to be an “at-risk person” meaning being unintentionally at risk. OTRs are constantly being exposed to the likelihood of graft rejection, and as a consequence, they are treated with immunosuppressive medication as long as the graft is in place.

Threat might be defined as “an expression of an intention to inflict pain, injury, evil, or punishment; an indication of impending danger or harm; one that is regarded as a possible danger; a menace” (The American Heritage Dictionary of the English Language, 2000, p. 1801). According to Lazarus and Lazarus (1994), it remains a threat when harm has not happened but is expected. Lazarus (1991) also defined threat as being “a threatening encounter that makes one feel uneasy (anxious), which is connected with a strong effort to protect oneself from anticipated danger” (p. 18). Furthermore Lazarus and Folkman (1984) described threat as “harms or losses that have not yet taken place but are anticipated. Even when harm/loss has occurred, it is always fused with threat because every loss is also pregnant with negative implications for the future” (p. 32). OTRs expect damage to happen if graft rejection occurs, that is, reduced function of their transplanted organ. Most of them make strong efforts to protect themselves from graft rejection, which we have reported in Nilsson et al. (2008), and about 33% of the OTRs fear that this will actually happen (Nilsson et al., 2011).

Furthermore, Carpenter (2005) argued that perceived threat is a threat based on a perception, and it is perception of some anticipated harm. The harm can be revealed in various forms such a perceived loss, interference with needs or goals, and perceived loss of control. It is the individual’s perception of the cue or event that is meaningful, not the kind or quality of the perceived anticipated harm. A reasonable assumption is that this perceived threat also involves various psychological reactions, such as efforts to cope with the perceived threats. This reasoning was evident when we explored the perceptions of experiences of graft rejection among OTRs (Nilsson et al., 2008).

According to Carpenter (2005), perceived threat can be defined as “the anticipation of harm that is based on the cognitive appraisal of an event or cue that is capable of eliciting the individual’s stress response” (p. 194). First, threat is based on a perception. This is important because of the fact that perceptions are culturally constructed and a function of one’s social environment and can be specific to the individual. Second, threat is based on the perception of anticipated harm. Third, perceived threat comes from a cognitive appraisal of an event or cue.

In the event of a perceived threat, the individuals’ perception of a threatening event is based on a cognitive appraisal to that event. What is appraised as threatening to one individual might be appraised as challenging to another. And finally, perceived threat exhibits itself as an emotional response that is part of an individual’s stress response. (Carpenter, 2005, p. 194)

### Concept and Statement Development

To test the concept, we performed a phenomenographic study aimed at exploring perceptions of experiences of graft rejection among OTRs receiving a kidney, liver, heart, or lung. This study provided an in-depth understanding of the patient perspective and confirmed the proposed concept definition in all its parts. These findings are reported in detail elsewhere (Nilsson et al., 2008) and are only summarized here. In conclusion, the risk of graft rejection was perceived as the everlasting present threat affecting everyday life. The threat was about fear of facing death, being as ill as before the transplantation, loosing health, and facing re-transplantation. The level of fear increased by the biopsy procedure and while waiting for blood sample results. The efforts to cope with the risk of graft rejection, that is, the anticipated harm varied and involved various efforts to protect oneself from this. As the test of the concept confirmed our definition, we suggest that PTRGR is defined as follows:

The anticipation of graft rejection is based on symptoms, signs or the cognitive appraisal of graft rejection. This anticipation is capable of eliciting the stress response of organ transplant recipients, expressed as intrusive anxiety and fear of negative health implications for the future. (Authors’ definition)

When testing the theory, we found that kidney transplant recipients reported significantly more graft-related threat than patients receiving a liver, heart, or lung. PTRGR was independent of time since transplantation, experienced graft rejections, and clinical differences among OTRs (Nilsson et al., 2011; Nilsson et al., 2013).

### Synthesis and Theory Development

In this phase, we used information from the literature review, as well as from our empirical observations to construct a new theory. The next step was to develop an instrument to measure PTRGR (PTGR-instrument) where we named the three factors in the factor structure: (a) graft-related threat, (b) intrusive anxiety, and (c) lack of control. The PTGR-instrument measures the concept by 12 items on a 5-point Likert-type scale (Nilsson et al., 2011). The categories from the previous phenomenographic study were now revised and psychometrically validated by a three-factor solution. The meaning of the first factor known as graft-related threat was a perception that the primary disease would return and one will be as ill as before the transplantation and facing re-transplantation. Thus, this factor showed the extent of the risk for anticipated harm and implications for the future. Regarding graft-related threat, the patients’ scores were widely spread, 33% of the patients perceived a low level of graft-related threat, 40% were uncertain, and 27% scored a high level of graft-related threat.

The second factor, intrusive anxiety, meant being constantly aware of the risk of graft rejection, thinking about it constantly. It also meant experiencing great anxiety, which was elevated when taking immune-suppressive medication or going through a biopsy. Thus, this factor showed the extent of the OTRs’ stress response and level of anxiety. The majority, 74%, of the OTRs scored low levels of intrusive anxiety.

Finally, the third factor, lack of control, involved perceptions that the threat by means of risk of graft rejection was out of one’s control, revealing the degree of belief that one can control and protect oneself from the threat. A high level of lack of control was experienced by 48%. The deductive stage of theory development began as a form of logical reasoning in which specific conclusions were inferred from more general premises, that is, the basic nursing concepts: person, health, environment, and nursing. The actual theory of PTRGR (PTRGR-theory) is proposed as follows.

### Major Concepts of the PTGR-Theory

We started to define the meta-concepts of nursing, as they appear in our theory. The person is an OTR, subjected to lifelong immunosuppressive medication because of a constant risk of graft rejection, to prevent the possible harm of graft rejection. The environment is any context where the OTR is trying to master his or her everyday life. Health is viewed as experienced comfort and cognitive understanding to master the PTRGR, resulting in an experienced well-being and HRQoL. Transplant nursing also involves the following:

- *Assessment*: Context-specific deliberative actions to approach and assess threat-induced emotions and actions to relieve intrusive anxiety in the OTR. Assessment can be either subjective or objective.
- *Development*: Person-centered nursing care plans, which is a standard intervention.
- *Implementation*: Context-specific threat reducing interventions that promote the OTRs’ mastering of graft-related threat and support adoption of useful and reasonable strategies to protect oneself from harm, that is, a graft rejection.
- *Evaluation*: The level of the PTRGR as a nursing outcome.

Subjective assessment starts with a one-on-one approach, listening to the OTRs’ perceptions of experiences of graft rejection. A simple introductory question might be the following: “When I say graft rejection, what comes to your mind”? The 12-item PTGR-instrument described above is useful for carrying out objective assessment. Repeated instructional conversations can be a useful intervention to promote the mastering of a graft-related threat. An important strategy to support the adoption of useful and reasonable strategies to protect oneself from harm is to assess barriers for adherence (Dobbels, Decorte, Roskams & Damme-Lombaerts, 2010). It is then possible to apply the pocket guide, developed by the International Transplant Nurses Society (ITNS; 2013), to provide an overview of the interventions that might be useful to overcome the different medication–adherence barriers experienced by OTRs. A sense of control over the medication regimen is expected to reduce the perceived threat of the fear of graft rejection. Finally, evaluation is performed, both subjectively and objectively in relation to the stated outcomes from the nursing care plan by interviews as well as the PTGR-instrument.

Additional major concepts of the PTRGR-theory are as follows:

- *Transplant care needs*—Occurs after discharge from the transplant unit when the OTR finds it difficult to adopt health-promoting behavior, accepting the new health situation, and remain adherent to immunosuppressive medication and the obtained self-care instructions. This concept was developed inspired by Kolcaba’s (2001) theory of comfort and her concept comfort needs. Transplant care needs are usually identified during follow-up in the outpatient transplant clinic.
- *Threat reducing interventions*—Context-specific deliberative nursing interventions, that is, assessment, development, implementation, and evaluation. The concept was inspired by the deliberative nursing process originally developed by Orlando (as cited in Faust, 2002).
- *Intervening variables*—Graft function, immuno-suppressive regimen and its possible side effects, health literacy, graft-related coping strategies, barriers for adherence, and social support. This concept was developed inspired by Kolcaba’s (2001) theory of comfort and her “concept intervening variables” as obstacles for comfort. In our theory, intervening variables are highly contextual, linked to the organ transplantation and established as important for long-term outcome after organ transplantation.
- *Protective strategies*—Coping strategies adopted by the OTR to protect oneself from harm caused by a possible graft rejection. These protective strategies were identified in one of our first studies (Nilsson et al., 2008).
- *Evidence-based practice*—Evidence-based standardi-zed nursing care plans, protocols, instruments, and procedures developed in transplant units for specific OTRs. This concept is highly relevant for transplant nursing, but general and important for all nursing practice regardless of context.

Our perspective of evidence-based practice stems from the model developed by Scott and McSherry (2008). The model shows that to obtain the best possible evidence, the supporting factors have to be used in a particular internal sequence, in which the first level includes national guidelines, policies, and empirical research. The second level comprises local policies, clinical experience, and nursing theories. The third level involves the use of practitioners’ knowledge and experience, which are critically evaluated with reference to Level 1 and Level 2, before continuing with Level 4, where the patient is involved in the decision making and evaluation of care.

At this point, we developed the following tentative framework and statement.

An addition, between a combination of transplant care needs, the threat reducing interventions and the intervening variables, leads to a reduced perceived threat, which in turn is affected by protective strategies. (Authors’ definition)

### Analysis and Theory Revision

**Figure 1.**
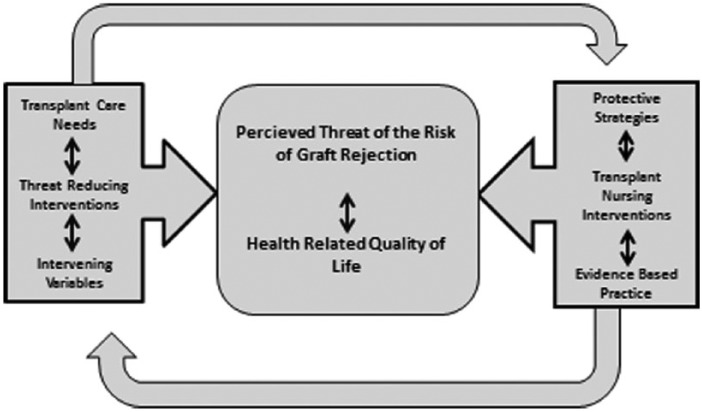
The framework of the Middle Range Theory of the Perceived Threat of the Risk of Graft Rejection and its inherent relationships.

When using analysis, we chose to dissect the whole, that is, the tentative framework, into its parts again, so that they could be better understood. We tested the theory by exploring relationships between the factors in the PTGR-instrument, psychological reactions to PTRGR and HRQoL as well as various clinically relevant variables as reported in detail in a previous study (Nilsson et al., 2013). Mainly positive coping strategies were used, that is, social trust, minimization, and problem reducing actions. Those having had a rejection experienced more isolation, women used more fatalism, and OTRs younger than 50 reported more intrusion. There was also a very strong relationship between the factors, lack of control, the coping strategy, and fatalism, confirming that lack of control is synonymous with a fatalistic approach. These coping strategies also gave consequences regarding HRQoL. The negative coping dimensions, isolation and protest, decreased general health, vitality, role-emotional, social functioning, and mental health; however, there was no relationship at all between the positive coping dimensions and HRQoL. Based on these findings and the previous steps in the process of theory building, the theoretical framework was revised resulting in Figure 1. Transplant care needs, threat reducing interventions, and intervening variables affect and will result in a certain level of PTRGR and HRQoL in everyday life. The level of PTRGR is affected by the OTRs’ use of protective strategies, transplant nursing interventions, and evidence-based practice performed by the transplant nurse. The level of PTRGR and HRQoL in everyday life mutually affects each other. The use of protective strategies and the nurse’s transplant nursing interventions and evidence-based practice will not only affect the level of PTGR and HRQoL, it will also affect the transplant care needs, the threat reducing interventions, and the intervening variables, which in turn affects the OTRs’ choice of protective strategies and the nurse’s choice of evidence-based practice.

**Figure 2.**
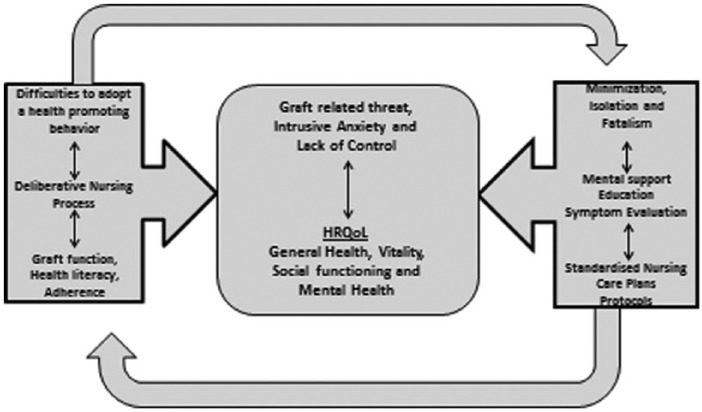
The content in the framework of the Middle Range Theory of the Perceived Threat of the Risk of Graft Rejection (PTRGR) and its inherent relationships.

Transplant care needs, threat reducing interventions, and intervening variables will result in a certain level of PTRGR and HRQoL in everyday life. This level of PTRGR is affected by and affects the OTRs’ use of protective strategies and evidence-based practice as well as transplant nursing interventions performed by the transplant nurse. The level of PTRGR also affects everyday life and HRQoL. The use of protective strategies and the nurse’s transplant nursing interventions and evidence-based practice will both affect the level of PTGR and the transplant care needs, the threat reducing interventions, and the intervening variables. The evidence-based practice strategy that is selected depends on the OTRs’ use of protective strategies. The content of the framework is illustrated in Figure 2. A transplant care need might be identified by the OTRs’ difficulties to adopt a health-promoting behavior because of the fear of graft rejection. The transplant nurses promote threat reducing interventions, that is, deliberative nursing process, and consider various intervening variables, for example, the graft function, the medication, and adherence. As a result, the level of PTRGR, that is, graft-related threat, intrusive anxiety, and lack of control might be affected. The relationship between the parts of PTRGR and HRQoL is established in research. It is also scientifically established that protective strategies, that is, minimization, isolation, and fatalism, affect HRQoL. When adding evidence-based practice, for example, standardized nursing care plans, to the patient’s protective strategies, the perceived threat and the HRQoL might be affected in a positive way.

This framework will serve as the proposed theory of PTRGR and will also serve as a foundation for further theory testing.

## Discussion

This is the first effort ever to propose a middle-range nursing theory useful in relation to OTRs facing the PTRGR. As such, this proposed theory has of course several limitations. First, not all relationships are tested in robust studies. Second, the effects of threat reducing interventions are not established. Instead, it is based on clinical assumptions and extensive clinical experience. The proposed intervening variables are validated by the literature review and clinical experience. Some of these variables are also established by research of symptoms and non-adherence (Forsberg & Lennerling 2012; Forsberg, Persson, Nilsson, & Lennerling, 2012). Third, it is directed toward and tested on adult OTRs excluding, for example, the vulnerable group of adolescents. The theory needs further testing but has reached sufficient clarity at this stage to be presented.

We have proposed that this MRT of PTRGR is more specific than a conceptual model (Meleis, 2007) because it is organized, coherent, and systematically articulates a set of statements related to the specific situation of PTRGR in transplant nursing. We have also tried to communicate this in a meaningful way, in its entirety, and to illustrate the framework and its internal relationships by the two figures (Figures 1 and 2).

The theory originally emerged while performing several research studies, reported in the “Introduction” section, and has taken shape stepwise by the use of Walker and Avant’s (2011) excellent steps of theory development. The purpose of this MRT of PTRGR is to describe situations, conditions, and relationships to assist transplant nurses who are (a) caring for OTRs suffering from threat-induced emotions that negatively affect and limit their everyday life and HRQoL and (b) detecting a risky protective behavior, that is, isolation, avoidance, or non-adherence to the immunosuppressive drugs.

The proposed theory is a conceptualization of the core aspects of OTRs’ reality that relates to transplant nursing and deals with several specific substantial concepts and propositions. We also argue that the framework might be referred to as a MRT because it has a narrow scope, is substantial and practical, and includes few concepts and propositions (Fawcett, 2005). It represents a limited or partial view of nursing reality, that is, transplant nursing, and is appropriate for empirical testing (Liehr & Smith, 1999) in its various parts as we have shown previously. Finally, we believe that the theory is applicable to clinical practice (Smith, 2008).

According to Chinn and Kramer (2008), the criteria for theory analysis should include clarity, simplicity, generality, empirical precision, and derivable consequences. By using both text and figures, we have tried to reach the criteria of clarity. There is a need for simplicity, and the described relationships are probably not difficult to understand for the initiated transplant nurse. When transplant care needs are approached by the nurse’s threat reducing interventions and intervening variables are considered, it will affect the level of PTRGR. We have also established that there is a strong relationship between PTRGR and reduced HRQoL (Nilsson et al., 2011; Nilsson et al., 2013) as well as limitations in everyday life. We know from our first phenomenographic study (Nilsson et al., 2008) that the OTRs use various protective strategies to affect the level of PTRGR. Some of these strategies are considered to be negative.

Finally, evidence-based standardized nursing care plans and protocols can affect the OTRs’ protective strategies. This demands new strategies so that the situation can be approached in a health-promoting manner. The impact of evidence-based practice has echoed across nursing practice, and nurses have responded to launch initiatives that maximize the valuable contributions that nurses can make to fully deliver on the promise of providing evidence-based practice to all patients in need of health promotion nursing interventions. Such initiatives include theory development. Nevertheless, many transplant nursing interventions are useful even if they lack the standard of evidence-based practice.

Regarding generality, this MRT is highly contextual. However, person-centered care, deliberative nursing interventions, and evidence-based practice are all essential and universal in nursing practice. The empirical precision has proven to be promising. We have already found that it is possible to test several links in the theory as well as validate the content of the concept of PTRGR. Finally, the importance of the theory is left for reviewers to judge; however, because of a total lack of MRTs offering guidance in the area of transplant nursing and the fact that PTRGR is highly relevant for OTRs, the theory might be of importance.

Other important aspects when discussing a theory are the factors of social utility, social congruence, and social significance. In respect of social utility, there might be a need for the transplant nurse to undergo brief training before clinical use. Social congruence considers whether the model will lead to nursing activities that will meet the expectations of the public. We assume that the MRT will inspire nursing practitioners to utilize several deliberative nursing activities that meet the expectations of the OTRs. The MRT will scarcely meet the criterion of social significance or make any difference in the health conditions of the public. Finally, we strongly believe that this MRT, despite its limitations, still offers much to the discipline of transplant nursing by increasing the understanding of the PTRGR.
